# Body image at the trunk: An investigation into externally referenced width perception and picture mapping

**DOI:** 10.1177/03010066241263052

**Published:** 2024-08-01

**Authors:** Simon Pratt, Benedict M. Wand, Dana A. Hince, Mervyn J. Travers, Lee Schneider, Sara Kelly, William Gibson

**Affiliations:** School of Health Sciences, 3431 The University of Notre Dame Australia, Fremantle, Australia; School of Health Sciences, 3431 The University of Notre Dame Australia, Fremantle, Australia; Institute for Health Research, 3431 The University of Notre Dame Australia, Fremantle, Australia; School of Health Sciences, 3431 The University of Notre Dame Australia, Fremantle, Australia; School of Health Sciences, 3431 The University of Notre Dame Australia, Fremantle, Australia; School of Health Sciences, 3431 The University of Notre Dame Australia, Fremantle, Australia; School of Health Sciences, 3431 The University of Notre Dame Australia, Fremantle, Australia

**Keywords:** Body image, metric measures, depictive measures, template matching, externally referenced width perception, body representation

## Abstract

Body image is a conscious representation of the body, encompassing how our body feels to us. Body image can be measured in a variety of ways, including metric and depictive measures. This study sought to assess body image at the trunk by investigating, and comparing, a metric and depictive measure. Sixty-nine healthy participants estimated their thorax, waist, and hip width by externally referencing mechanical calipers. Participants were also asked to select the true image of their trunk from a random display of nine images containing the true image and incrementally shrunken or enlarged images. Participants demonstrated evidence of thorax and waist width overestimation in the width perception task, with no evidence for hip misestimation. For the picture mapping task, the majority of participants were inaccurate. In participants who were inaccurate, approximately equal proportions underestimated and overestimated their trunk width. The two tasks were found to be independent of each other. Distortions, or inaccuracies, were apparent in a metric measure, and inaccuracies also present in a depictive measure, of body image at the trunk for healthy participants. An overestimation bias was apparent in the metric, but not depictive, task. No relationship was found between tasks..

Body image is a conscious representation of the body, and refers to how our body *feels* to us (Levenig et al., 2016; Lotze & Moseley, 2007). It is a perception of *what* our body is (Longo et al., 2010), and is the conscious development and maintenance of the sense of self (Longo, 2015a; Longo et al., 2010). Therefore, body image is the feeling of the body size, shape, and physical composition that is consciously experienced (Longo, 2022). Body image encompasses the subjective perception of the physical appearance of one's own body and the associated feelings with this (Levenig et al., 2016), and provides a description of how body parts relate to each other, and the functional purpose of body parts (de Vignemont, 2010). The dynamics of body image ranges from short-term body perception to long-term body beliefs and can be used for one's own body and for someone else's body (de Vignemont, 2010). In addition, body image can be modulated by memory, belief, and psychosocial factors (Lotze & Moseley, 2007). Body image can be classified into perceptual body image under the term of “somato-perception” or in a cognitive-affective body image domain, under the term “somatorepresentation” (Levenig et al., 2016; Longo, 2015a, 2016; Mölbert et al., 2017).

There is evidence to suggest body image is disrupted in pain, with previous studies reporting participants with pain demonstrated altered perceived hand size with a hand image mapping task (Gilpin et al., 2014; Moseley, 2005; Peltz et al., 2011), altered body drawing of the lumbar spine (Moseley, 2008) and neck (Moreira et al., 2017) and altered self-perception via questionnaire responses (Wand et al., 2014, 2016, 2017). Body image, therefore, could be viewed as a potential target for intervention for patients with chronic pain. For this reason, researchers require methods of assessing body image and normative data for comparisons.

This study focuses on perceptual measures of body image with body width estimation tasks (Mölbert et al., 2017), utilizing a metric measure and a depictive measure (Longo & Haggard, 2012). In a metric measure of body image, participants compare the size and shape of their body to a standard nonbody object such as a set of calipers or a ruler (Longo & Haggard, 2012; Mölbert et al., 2017). For a depictive measure of body image, participants compare their bodies to a visual depiction of a body (Longo & Haggard, 2012). For both types of tasks, researchers are interested in investigating the characteristics of differences between perceived and actual body size.

The current study utilized a metric measure, with participants estimating the distance between mechanical calipers for the width of their thorax, waist, and hips, and a depictive measure, where participants performed a picture mapping task, aiming to select the true image of their trunk from a selection of images that have been enlarged or shrunk. To our knowledge, this is the first study to conduct these body image measures in this manner in the same testing session for the trunk. The aims of this study were to establish normative values of the difference between perceived and actual for a metric and depictive measure of body image at the trunk and explore the relationship between these two measures.

## Methods

### Participants

This cross-sectional observational study was conducted on healthy individuals within a university research laboratory. The study was approved by the Institutional Human Research Ethics Committee (Reference Number: 017188F). All participants provided signed informed consent and all procedures conformed to the Declaration of Helsinki.

A consecutive sample of 69 healthy volunteers was recruited via advertisement and word of mouth from The University of Notre Dame Australia and the local community between February 2018 and March 2019. Inclusion criteria were: aged 18–60; currently low back pain free; no history of low back pain lasting more than 24 hr within the last six months; no low back pain requiring medical attention within the last two years; no other significant musculoskeletal pain (>1/10); able to stand in a stable position for up to 1 hr; proficient in written and spoken English; and able to provide informed consent. The inclusion criteria relating to the ability of the participant to stand in a stable position for up to 1 hr was included as this study was part of a wider project where participants needed to stand for prolonged periods. Exclusion criteria were: known body perception difficulties (e.g., body dysmorphic disorder; anorexia; vestibular disorder); noncorrectable visual impairment; unstable balance in standing; any current neurological, musculoskeletal, or widespread pain disorder; any significant existing medical condition; and any large tattoo over the back which could not be suitably erased on the digital photo.

### Experimental Procedures: Externally Referenced Width Perception Task

For the externally referenced width perception task, a set of calipers (Mentone Educational Anthropometer Measuring Set) with the precision of 1 mm was used. Each participant was provided with an explanation of the procedure and shown a body outline image to orientate them to the different body regions being tested. The explanation was as follows:

This task involves you estimating how wide you are at three different levels of the body. We will ask you to stand with the arms away from the side of your body, and the sheet lightly pegged around your neck.

This diagram explains the terms we use for each of the three different body regions. It is important to note that when estimating the size of your thorax, this does not include your shoulders, it is just from one side of the chest to the other.

I will adjust these movable calipers, and I will either be moving the calipers from far apart to closer in, or from together to far apart. I will perform this at three different levels. I would like you to tell me:

- how wide you are at the widest part of your thorax
- how wide you are at the narrowest part of your waist
- how wide you are at the widest part of your hips.

Participants were then given a demonstration of how the calipers could be moved. Participants next performed a practice trial with the calipers for familiarization.

For formal testing, participants were asked to stand in a standardized position with their feet behind a line marked on the floor and had a black sheet placed over their body secured loosely around their neck, so they could not see their body. As mentioned in the instructions, participants were asked to keep their arms out from the side of their body, to avoid their arms touching their trunk.

**Figure 1.**
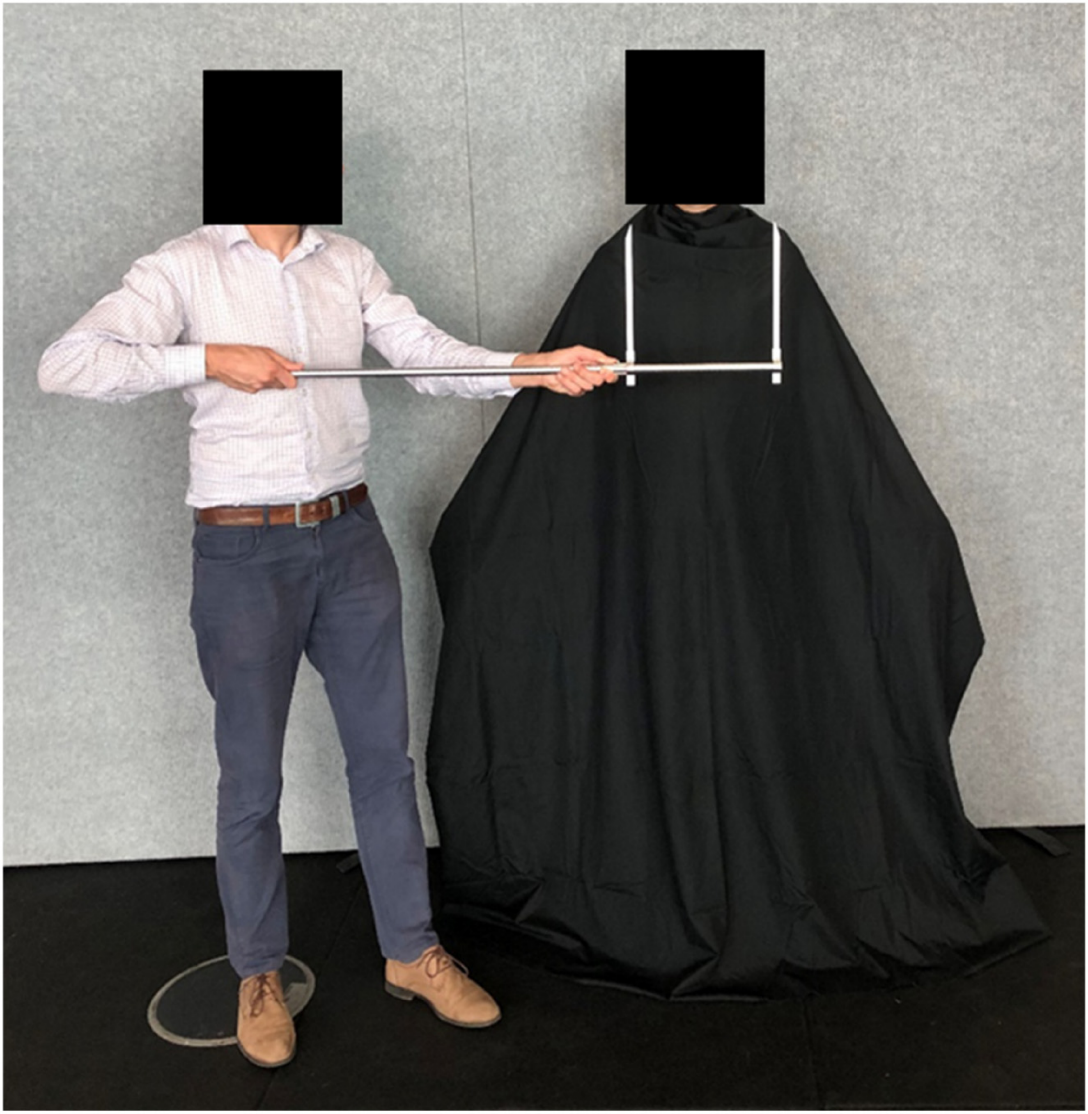
Externally referenced width perception task using adjustable calipers.

The calipers were held in front of the participant by the researcher with the measurement numbers on the caliper facing away from the participant. The right-sided vertical arm of the caliper was held in line with the right edge of the participant's trunk and the horizontal arm was positioned in line with the body area being assessed (Figure 1). The areas assessed corresponded to three body levels: the widest part of their thorax, the narrowest part of their waist, and the widest part of their hips. The researcher slowly moved the caliper arms, and the participant instructed the researcher to stop at the point at which the caliper distance corresponded to their perception of body width at the level being measured. Participants were then asked to keep their eyes facing forward toward the wall while the examiner recorded the value. The vertical arms of the caliper were either started together and moved slowly apart by the examiner (in-to-out) or started wide apart, and moved slowly together (out-to-in), with each variation performed twice at each body level (12 tasks in total). The order of task performance was determined by random allocation.

Following the performance of all 12 tasks, the black sheet was removed, and the examiner stood behind the patient and used the caliper to make actual measurements of the widest part of their thorax, the narrowest part of their waist, and the widest part of their hips. These values were used to compare against the participant's perceived measures for data analysis. The full assessment procedure was repeated on two separate occasions, approximately 40 min apart. These data were collected to assess the reliability of this task (see Appendix A). Due to a data entry error with the first trial, the first set of 12 tasks was deemed a practice trial, and only data from the second set of 12 tasks were used for analysis.

### Experimental Procedures: Picture Mapping

**Figure 2.**
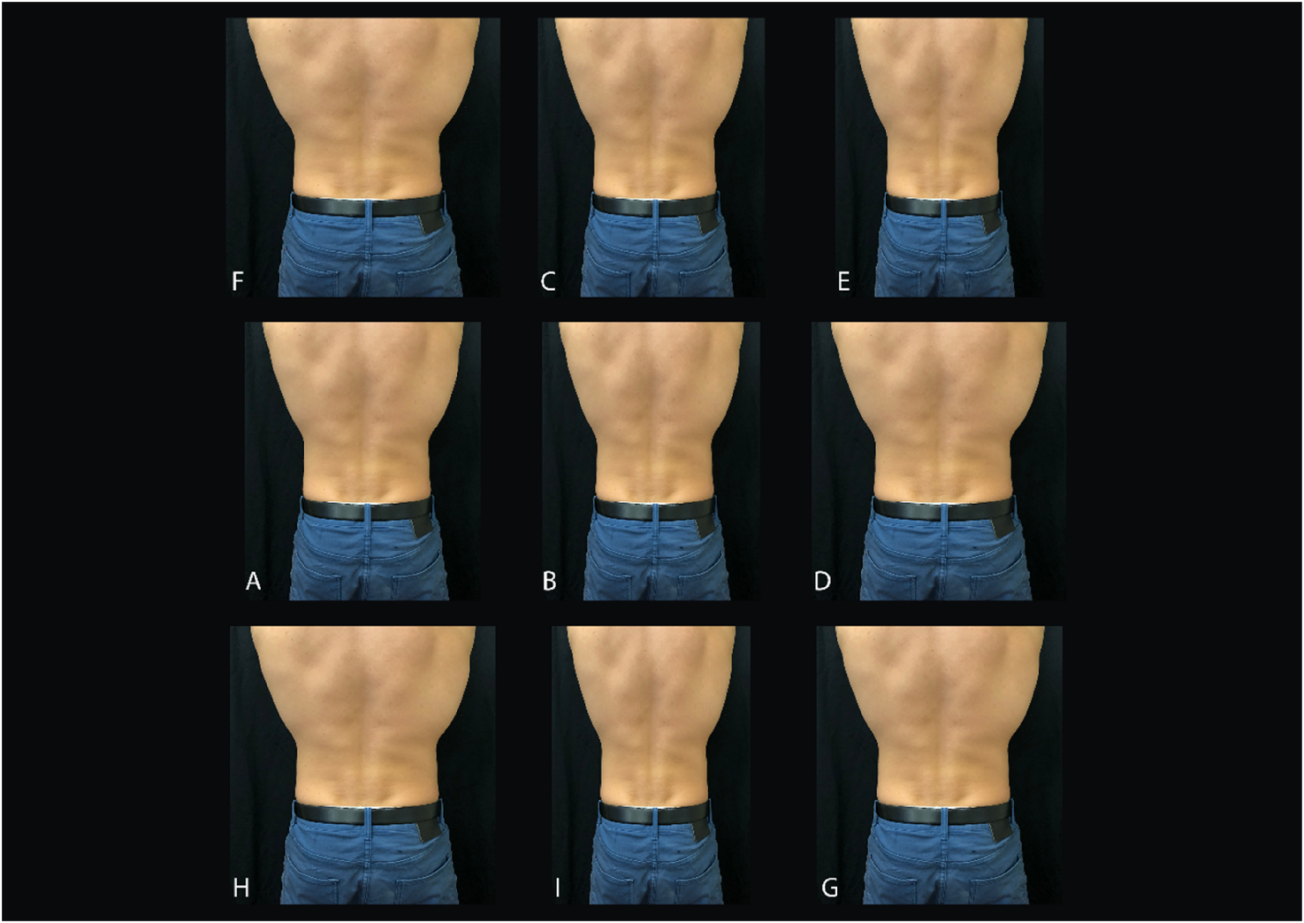
Picture mapping task images as per presented to participants.

For the photo, the participant removed their tops, aside from underwear, and crossed their arms across the front of their chest, to avoid their arms appearing in the photo. With the participant standing facing a wall lined with a black sheet, a digital photo of the participant's trunk was taken with an Apple iphone8. The image was then imported into Adobe Photoshop (CC 2017). In Photoshop, the image was cropped at the level of the spine of the scapula superiorly, the gluteal fold inferiorly, and laterally at equal distances just beyond the outside border of the widest part of the participant's trunk. This standardized cropped image was then manipulated within Photoshop using a preprogrammed syntax file which produced nine images, arranged in a randomly generated order. One image was not altered (true image), four images were shrunk in width (84%, 88%, 92%, 96%), and four images were enlarged in width (104%, 108%, 112%, 116%). Images were adjusted in size only by width, and not by height. The sheet of images was then color printed on A3 paper (Figure 2).

The participant was seated in front of the printed sheet and provided with the following instructions:

The following images contain the true picture of your back, and images which have been enlarged in size, and images which have been shrunk in size. I would now like you to select from the following images, the picture you believe to be the true image of your back.

The image selected was recorded and the percentage magnification was noted. Previous researchers have found this test reliable when used for the hand, with an intercorrelation coefficient (ICC) > 0.93 (Moseley, 2005), and when used for body frontal and profile images, with retest reliability 0.90 for frontal estimates, and 0.86 for profile estimates (Freeman et al., 1984).

### Data Analysis

Statistical analysis was performed using the Statistical Package for Social Sciences (SPSS) version 24 (IBM Corporation, New York, USA), and STATA- V17 (StataCorp. 2017. *Stata Statistical Software: Release 17*. College Station, TX: StataCorp LLC). The demographics of participants were summarized with means and standard deviations (SDs) for continuous data and frequency/percentages for categorical data.

The main outcome measure for the externally referenced width perception task, previously used similarly for analysis of size estimation tasks, was the body perception index (BPI) (Ben-Tovim et al., 1979; Slade & Russell, 1973; Smeets et al., 1998), defined as the perceived width divided by the actual width, multiplied by 100, for each trial at the three body levels. Other studies have also used similar approaches for the analysis of body size and landmark estimates (Linkenauger et al., 2015; Longo & Haggard, 2010). A BPI of 100 indicated perfect accuracy when comparing perceived width to actual width. A BPI greater than 100 indicated the perceived width was larger than the participants’ actual width (i.e., the participants perceived themselves as wider than they actually are). A BPI less than 100 specified the perceived width was smaller than the participants’ actual width (i.e., the participants perceived themselves as thinner than they actually are). A linear mixed model was fitted to the BPI to investigate whether the BPI varied with body level. Random effects included in the model were caliper movement (in-to-out or out-to-in) crossed with level, and nested within participant.

For the picture mapping task, the question of whether participants were accurate or not was considered in two ways by binomial test. First, we considered true accuracy vs not accurate (100% category choice vs all others) by comparing the proportion of responses in the “accurate” category to that expected by chance (i.e., 1/9 or 11%). Second, the proportion of inaccurate responses that underestimated trunk width was compared to that expected if only chance were a factor (i.e., 1/2 or 50%). Moreover, 95% confidence intervals (CIs) were calculated for all proportions using the Clopper–Pearson exact method.

To explore if there was a relationship between the two body image measures, linear mixed models were again fitted to the BPI, with the nine-category picture mapping variable (as a continuous predictor) and the three-category picture mapping variable (accurate/underestimation/overestimation) included as a fixed effect in two separate models. The same random effects as described above were also included.

The mixed models were assessed graphically to ensure conformity to assumptions of homoscedasticity, normality of residuals, and random effects. Recorded values for individual trials that produced residuals >5 standard deviations from the mean were investigated individually for error. If it was clear the recorded value was not possible or showed evidence of human error, the value for that trial was set to missing. All model-estimated means are presented with 95% CIs and *p* < 0.05 was considered evidence for a difference.

The data used in this study was collected as part of a broader study, and so no a priori sample size calculation was conducted for these analyses. Sixty participants per group provided over 80% power to detect a between-group difference in horizontal direction bias at the midline of 17.5 mm (SD = 34 mm) when compared with a low back pain group of the same size at the alpha = 0.05 level, however this analysis is not reported here.

## Results

Ninety-eight participants were screened for eligibility with 29 excluded for the following reasons: history of low back pain within the last six months (12); low back pain requiring medical attention, or lumbar spine surgery, within the last two years (1); known body perception difficulties (1); other musculoskeletal or widespread pain presentations (12); significant existing medical condition (2); and large tattoo over the back (1). Sixty-nine healthy participants were enrolled, including 34 males (49.3%) and 35 females (50.7%). Participants had an average age of 29.3 years (standard deviation [SD] = 9.5), and an average body mass index of 23.9 (SD = 3.8). Sixty-three (91.3%) participants were right-handed, and six (8.7%) were left handed. For the externally referenced width perception task, all 69 participants completed all tasks, with no missing data. For the picture mapping task, data from four participants were excluded due to large legible writing on underwear (n = 3) and a tattoo (n = 1) which revealed the size manipulation of the image. This tattoo had not been detected in the screening process and was too large to be digitally obscured. From this point onward, an additional screening process was added to screen participants for tattoos, and if tattoos were too large to be digitally obscured on the photo by Photoshop, participants were excluded from testing.

### Externally Referenced Width Perception Task

BPI values for the thorax, waist, and hips can be found in Table 1. Body level assessed was related to the mean BPI (effect of level, χ2 [2] = 145.88, *p* < 0.001). There was evidence for overestimation of width at the thorax and the waist, with the larger overestimation at the waist. There was, however, no evidence of misestimation at the hips (Table 1). The mean BPI at the waist was 8.1 percentage points greater than the thorax (95% CI = 5.6 to 10.6, *p* < 0.001) and 15.4 percentage points greater than that observed for the hips (95% CI = 12.9 to 17.9, *p* < 0.001). The mean BPI at the thorax was 7.2 percentage points greater than that at the hips (95% CI = 4.7 to 9.7, *p* < 0.001).

**Table 1.** Externally referenced width perception task: mean body perception index (BPI) (%), associated 95% confidence interval (CI), and *p*-value for the thorax, waist, and hips.

Body level	Mean body perception index (BPI) (%)	95% Confidence interval (CI)	*p*-Value^a^
Thorax	109.6	106.9 to 112.4	<0.001
Waist	117.8	115.0 to 120.5	<0.001
Hips	102.4	99.6 to 105.2	0.091

a Wald χ^2^ test for body perception index = 100% (i.e., perceived equals actual).

When assessing for differences between males and females, the body level assessed was related to sex (effect of level, χ2 [2] = 22.79, *p* < 0.001). Compared with males, for females, the BPI was 7.0% higher at the thorax (95% CI = 1.7 to 12.3, *p* = 0.009), 10.6% higher at the waist (95% CI = 5.3 to 15.9, *p* < 0.001), yet nonsignificant and only 0.5% smaller at the hips (95% CI = -5.8 to 4.8, *p* = 0.849).

The externally referenced width perception task was found to be a reliable measure with good-to-excellent ICC point estimates with the lower bounds for the confidence intervals for these in the moderate-to-good range, and with SEM values, expressed as a percentage of the perceived minus actual mean, varying between 33.3% and 97.8% (See Appendix A).

### Picture Mapping Task

**Figure 3.**
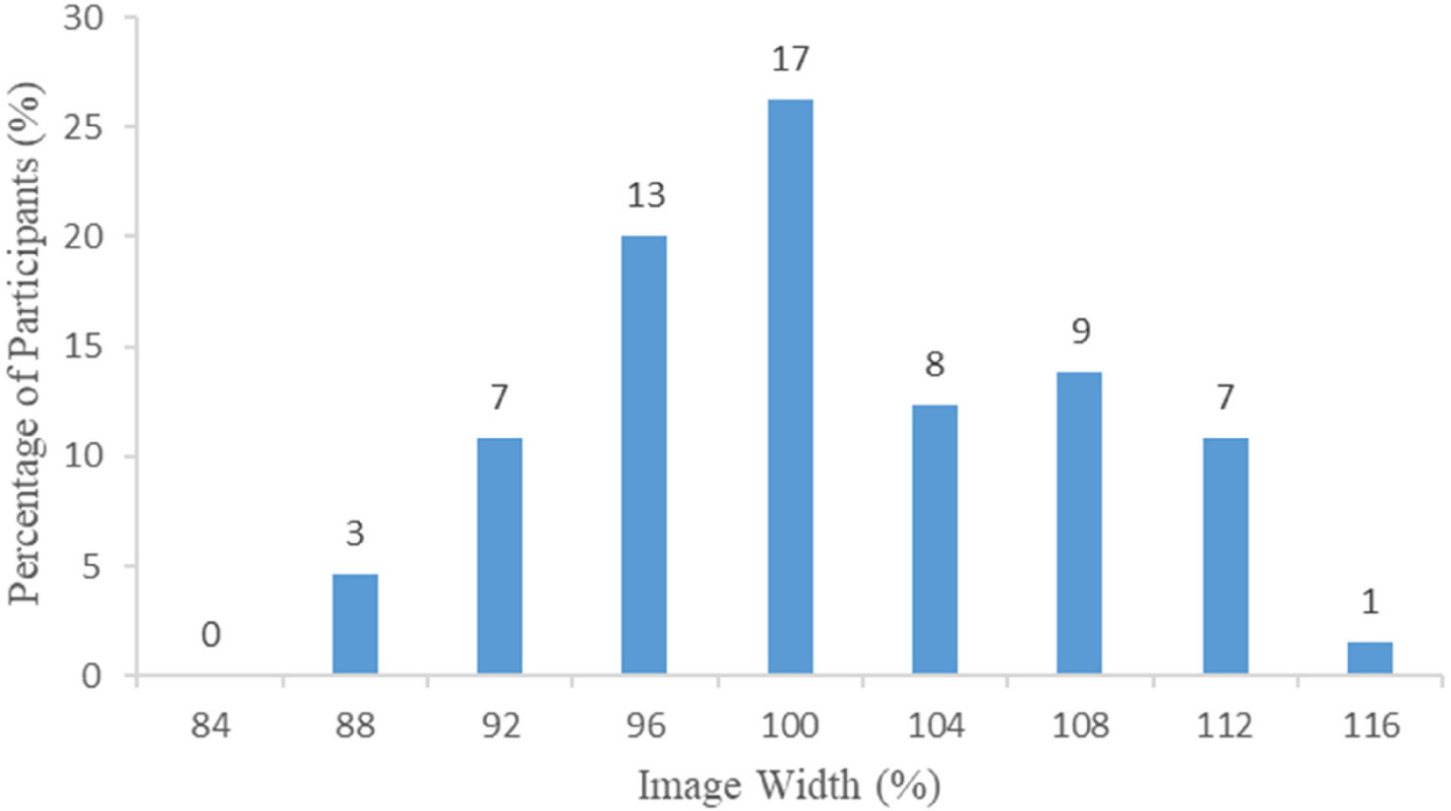
Picture mapping task: the percentage of participants selecting each image, by image width. The numbers above the bars represent the number of participants selecting each image width.

Figure 3 presents the proportion and number of participants choosing each of the nine picture image width categories. The accurate (100%) image was the most selected with just over a quarter of participants choosing this option. In general, the proportion of participants choosing each category decreased as a function of the degree of distortion (see Figure 3; proportion and 95% Confidence Intervals are also presented in Appendix B: Table A2).

As there were nine potential options for the participant to choose, we would expect one in nine (11%) to be accurate if selection was based only on chance, that is, approximately seven participants would select the accurate image. Seventeen participants were accurate, which corresponds to a 26% (95% CI = 16% to 39%) success rate, with the probability of observing a success this high being unlikely if the correct choice was due to chance (*p* < 0.001). Seventy-four percent (95% CI = 61% to 84%) of participants were inaccurate, with approximately equal proportions underestimating (48%, 95% CI = 33% to 63%) and overestimating (52%, 95% CI = 37% to 67%) their trunk width, with no evidence of systematic selection of under- or overestimation (*p* = 0.885). Further analysis revealed no relationship between sex (male and female) and being accurate or not (Fisher's exact: *p* = 0.096), and under or overestimating (Fisher's exact: *p* = 1.000).

### Relationship Between Externally Referenced Width Perception and Picture Mapping Tasks

**Figure 4.**
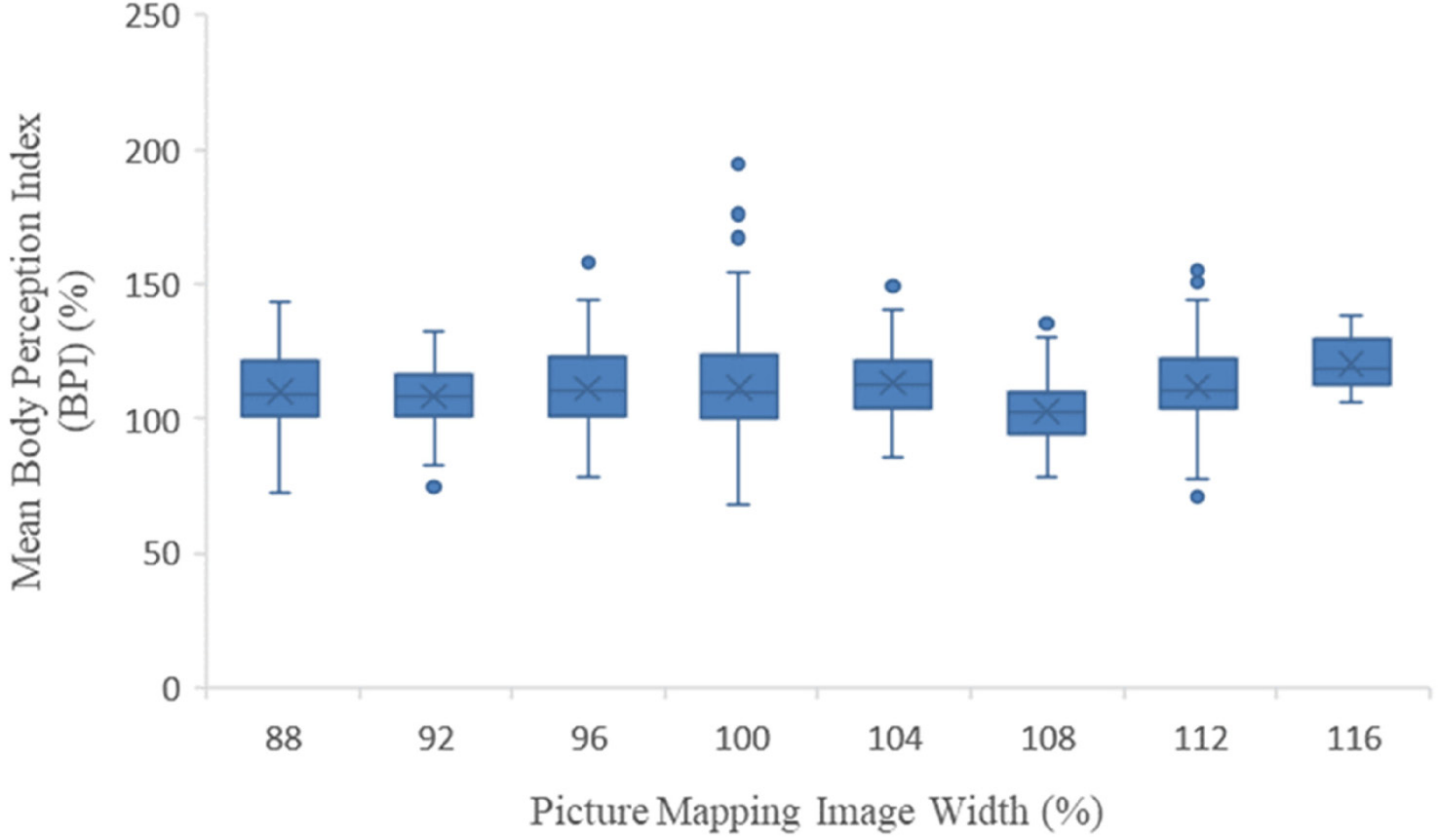
Box and whisker plots of mean body perception index (BPI) (%) verses picture mapping image width (%) selected.

No relationship was evident between the mean BPI and image width category chosen in the picture mapping task (slope of the line of best fit = −0.1, 95% CI =−1.6 to 1.4, *p* = 0.892) Figure 4.

Furthermore, no association was evident between mean BPI and whether the participant was accurate, underestimated, or overestimated in the picture mapping task (χ2 [2] = 0.46; *p* = 0.793).

## Discussion

The aims of this study were to investigate the metric and depictive measure of body image at the trunk, establish normative values of the difference between perceived and actual, and analyze if a relationship exists between these two measures. The metric measure (externally referenced width perception) involved participants estimating the distance between mechanical calipers for the width of their thorax, waist, and hips. For the depictive measure (picture mapping), participants were asked to select the true image of their trunk from a selection of images that had been enlarged or shrunk. For the externally referenced width perception task, participants overestimated the width of the thorax and waist, with a larger overestimation at the waist (17.8%) than the thorax (9.6%). No evidence was apparent for misestimation at the hips. When comparing the three body levels to each other, there was a greater overestimation of width perception at the waist compared to the thorax and hips, and the thorax compared to the hips. Furthermore, females were found to have a 7.0% and 10.6% higher BPI at the thorax and waist respectively. For the picture mapping task, the majority of participants were inaccurate with the task, however participants were more likely than chance to select the accurate image. For those participants who were inaccurate, approximately equal proportions underestimated and overestimated their trunk width. With analysis of each different width image, except for the image width of 108%, the number of participants selecting an image decreased as the image width became more shrunken or enlarged. No relationship was found between sex (male and female) and being accurate or not, and under or overestimating. The externally referenced width perception and picture mapping were shown to not be related to each other. Interestingly, whether a participant was accurate, underestimated or overestimated the picture mapping image, did not influence their mean BPI. It appears, therefore, that these two measures of body image may not be related.

Several methodologies have been employed to investigate the metric measure of body image and a number of body areas assessed. Participants estimating how different parts of their hand compared to the length of a line (Longo & Haggard, 2012; Tamè et al., 2017) were found to underestimate finger length (Longo & Haggard, 2012; Tamè et al., 2017) and overestimate hand width (Tamè et al., 2017), while right-handed participants who instructed an examiner to adjust a tape measure to match the perceived length and width of their hands were found to accurately perceive the size of their right hand but underestimate the size of their left hand (Linkenauger, 2009). Using the same methodology to estimate arm length, participants were found to accurately estimate the length of both arms if left handed but only accurately perceive their right arm, and underestimate their left arm if right-handed (Linkenauger, 2009). At the torso, as defined from shoulders to hips, participants estimating their length relative to a noncorporeal object (stick or dowel) were found to overestimate length (Sadibolova et al., 2019), while another study reported no such distortion (Linkenauger et al., 2015). The current study, measuring width and not length, also reports an overestimation of the torso's perceived dimensions.

Similar to the methodology of the current study, several studies adopted a moving caliper technique as a metric measure to estimate body width, with participants required to move two lights on a bar until it corresponded to their perceived width for their chest, waist, and hips (Dolan et al., 1987; Gleghorn et al., 1987; Slade & Russell, 1973), or adjust light beams to their estimate of width at their waist, hips, and thighs (Thompson & Spana, 1988). Consistent with our findings of overestimation at the thorax and waist, previous studies have shown male and female participants overestimated width at the chest (Males: 22.9%; Females: 24.2%), waist (Males: 20.2%; Females: 27.9%) and hips (Males: 10.3%; Females: 15.6%) with no evidence of difference between the male and female findings at each level (Dolan et al., 1987), and female participants overestimated width at the waist (35%), hips (17%), and thighs (11%) (Thompson & Spana, 1988). It is worth noting though that this is not a uniform finding; in an earlier study, despite still demonstrating a (slight) overestimation of waist width (0.2%), healthy female participants were found to underestimate their chest (5.0%) and hips (3.4%) width (Slade & Russell, 1973), consistent with another study reporting healthy female controls underestimated width at the shoulder (18.8%) and hip (4.7%), and overestimated waist width (4.1%) (Gleghorn et al., 1987). The current findings do however add weight to the theme of overestimation of the waist using metric measures.

Several studies have investigated body image using depictive measures. Longo and Haggard (2010) presented participants with a range of 15 images consisting of an average-looking hand (1 image) and distortions of this image stretched in length or in width by 5–35%, and asked participants to select the image most closely representing their hand, a so-called ‘template-matching task’ (Longo & Haggard, 2010), a methodology similarly applied by further studies at the hand (Longo, 2015b; Longo & Haggard, 2012) and for the whole body with differing hip width/height ratios (Fuentes et al., 2013a, 2013b). In these studies, participants were approximately accurate for the template-matching and image-matching tasks in the hand (Longo, 2015b; Longo & Haggard, 2010, 2012) and the whole body (Fuentes et al., 2013a, 2013b). In a task where female participants adjusted an image of their body to the estimated body size, results across body parts were very accurate, with only a tendency to overestimate body size of 1.9% (Tovée et al., 2003). These results suggest individuals appear to have a veridical conscious representation of hand and body shape (Fuentes et al., 2013a, 2013b; Longo, 2015b; Longo & Haggard, 2010, 2012; Tovée et al., 2003), implying an undistorted representation of body image. However, there is again ambiguity in these depictive measure findings, with results on a leg template-matching task demonstrating participants tended to overestimate leg width by approximately 10% (Stone et al., 2018) and results on a hand size estimation task also revealing overestimation of length with healthy participants (107.8%) (Gilpin et al., 2014). On a back template-matching task, where the back was enlarged or shrunk at the L4 level in increments of 3%, healthy controls also tended to select enlarged images (Meier et al., 2021). This is largely consistent with an earlier study, where participants adjusted distorted photographs of themselves in frontal, profile and back views to reflect their perceived body image, finding males and females—except for males’ back pictures—overestimated the stoutness of their bodies (Urdapilleta et al., 2010). Interestingly, when considering the dorsum and palmar aspects of the hand, D’Amour and Harris (2020) found participants perceived their dorsal hand longer than it actually was, but were quite accurate in judging the size of their palmar hand. These results suggest differing parts of the body may demonstrate distortions with depictive measures. Such distortions are certainly in line with the current study, with the majority of participants inaccurate with the picture mapping task. Intriguingly, when comparing these studies assessing depictive measures, predominantly, participants were approximately accurate when comparing their bodies to nonactual images of the participants’ bodies (Fuentes et al., 2013a, 2013b; Longo, 2015b; Longo & Haggard, 2010, 2012), whereas participants overestimated when comparing their body to an actual image of their body (Gilpin et al., 2014; Meier et al., 2021; Stone et al., 2018). Although this effect was not observed in our study, further studies could consider whether the use of the actual body image for the task influences results.

When considering depictive tasks in patients with chronic pain, overestimation of 107% (Moseley, 2005) and 108.6% (Peltz et al., 2011) was noted in patients with CRPS performing a hand matching task, despite healthy controls performing approximately accurately with the task (Peltz et al., 2011). However, on a hand matching task, patients with painful hand osteoarthritis, despite being fairly accurate in their hand length estimation (99.8% of real hand length), were significantly lower in their length estimation compared with the healthy control group (107.8% of real hand length) (Gilpin et al., 2014). Furthermore, on a back-photo assessment, participants with chronic low back pain tended to select enlarged images, with no meaningful between-group difference compared with healthy controls (Meier et al., 2021).

From the majority of the above findings, there appears a dissociation between depictive and metric measures, with participants mainly demonstrating accuracy with depictive measures, yet showing distortions with metric measures. This is in line with findings from the systematic review by Mölbert et al. (2017) comparing healthy controls to patients with anorexia nervosa or bulimia nervosa, highlighting healthy people were accurate in depictive methods yet overestimated in metric methods. In a systematic review by Tagini et al. (2021) comparing healthy controls to people who were overweight, healthy controls also overestimated when performing a metric task with visual size estimation, however, when performing a depictive measure task, healthy controls-although more accurate- also mainly overestimated in the task. Findings from our study also confirmed the two tasks were independent of each other, with inaccuracies in both tasks.

Seeking to explain this dissociation between depictive and metric measures, some authors suggest metric measures of body image may not be pure measures of the visual body image; in addition to its connection to explicit body representations, it may also exhibit a connection to somatosensory or implicit body representations, with depictive tasks more a pure measure of the conscious body image or explicit body representation (Longo & Haggard, 2012; Tagini et al., 2021).

The inaccuracies of participants in the current study with the metric task are consistent with previous findings of distortions with this task. However, the inaccuracies seen with the picture mapping task (depictive measure) appear largely inconsistent with the previously discussed findings of overall accuracy in these tasks with healthy participants. Perhaps the differences in our findings could in part be explained with this depictive measure utilizing a width adjustment of the participant's actual trunk as opposed to a nonactual image. Previous research used increments of 5% for the template mapping task (Longo & Haggard, 2010); perhaps the 4% increments used in our study were too close; however, this would not explain all the inaccuracies, as—although more concentrated around the accurate width image—there was a spread across most of the image widths. The previous study on back-photo assessment, where participants tended to overestimate their image and with the majority of participants inaccurate with the task, utilized increments of 3% (Meier et al., 2021). Their findings may also suggest the increments were too close or could indicate participants in general are not accurately estimating certain parts of their body, such as their back.

The findings in this study need to be considered in light of the limitations in the current study. For the externally referenced width perception task, only data for the second set of 12 tasks was used which meant participants were more practiced in the task and potentially did not capture participant's initial responses. The method of limits was used for the width perception task, where patients had to notify their width with either an increasing or decreasing size between the calipers. However, this method could be argued to lack precision as opposed to a staircase procedure, or the method of constant stimuli where participants could select from presented widths. Our data suggests that around 16% change is an appropriate limit for the picture mapping task as no participant chose the 16% shrunken image and only one participant chose the 16% enlarged image. However, a more fine-grained evaluation of width perception in this range might be more informative. Future investigations could consider using 3% increments to display 10 distorted images in the −15% to +15% range. In addition, as the picture mapping task was a perceptual measure of body image, the word “perceive” would have been better used instead of “believe” in reference to the instructions given to the participants where they were to select “the picture you *believe* to be the true image of your back.” Consequently, the use of belief rather than perception as the basis for judgment may have an implication for the results recorded. Furthermore, to add robustness to the results of the picture mapping task and then to be able to comment if distortions are present, a combination of further manipulation of trunk length as well as width and presentation of multiple sheets of the same altered images arranged in a different order could be used. The addition of further trials for this task could then enable analysis that also involves a differentiation between constant error (or bias) and variable error (or precision). Finally, the inclusion of clothing over the pelvis in the picture may provide some prompting as to width adjustment (a participant may be aware of the width of their clothing when flat), and so future studies could look to address this.

### Conclusion

This study assessed body image at the trunk via a metric measure using calipers for width perception of the trunk, and a depictive measure using a picture mapping task. In the metric task, participants displayed evidence of an overestimation of width at the thorax and waist, with the greatest overestimation at the waist. In the depictive task, the majority of participants were inaccurate, however, participants did choose the nondistorted image more than would be expected by chance. For participants who were inaccurate with this depictive task, approximately equal proportions underestimated and overestimated their trunk width. Performance in the two tasks was shown to be independent of the other. Distortions, or inaccuracies, appear evident at the trunk for a metric measure of body image, with inaccuracies also present when assessing a depictive measure.

## Appendix A: Externally referenced width perception test–retest reliability

### Data Analysis

To check for the reliability of the externally referenced width perception task, 30 participants were randomly selected from a sample of participants from this study (*n* = 14 healthy, 47%) and from a larger separate study of individuals with lower back pain (*n* = 16, 53%).

The mean width value at each level for each individual was calculated across the four trials at time 1 and the four trials at time 2. Intraclass correlation coefficients (ICCs) for the average were calculated using 2-way mixed models fitted to these means, with a random effect for ID and a fixed effect for the test. Standard error of measurement (SEM) was also calculated. Analysis was applied separately to the three body levels (thorax, waist, and hips) resulting in three assessments of reliability.

In accordance with previously published analysis, ICC values were classified with values less than 0.5 indicating poor reliability, values between 0.5 and 0.75 indicating moderate reliability, values between 0.75 and 0.9 indicating good reliability, and values greater than 0.9 indicating excellent reliability (Koo & Li, 2016).

SEM was calculated using the formula $SEM_{agreement}=\sqrt{\sigma_{retest}^{2}+\sigma_{residual}^{2}}$ (de Vet et al., 2006). This formula includes the variance attributed to the difference between testing sessions and the residual variance of scores. To calculate the variance components $\sigma_{retest}^{2}$ and $\sigma_{residual}^{2}$ we used the mixed command in Stata and considered the testing sessions and participants as random factors.

### Results

Table A1 displays the ICCs and associated 95% CI and SEM values for the reliability analysis of the externally referenced width perception task. Results indicate the externally referenced width perception task to be a reliable measure. The point estimate of the ICC suggests good-to-excellent test–retest reliability and the lower end of the 95% confidence intervals suggesting moderate-to-good reliability. SEM values, expressed as a percentage of the perceived minus actual mean, were found to lie between 33.3% and 97.8%.

**Table A1.** Mean difference (time 1 minus time 2), ICC, and SEM values for the externally referenced width perception task.

Body level	Mean difference for time 1 minus time 2 (mm) (95% CI; *p*-value)	Intraclass correlation coefficient (ICC) (95% CI)	Standard error of measurement (SEM) (mm)	Standard error of measurement (SEM) as a percentage of perceived minus actual mean (%)
Widest part of the thorax	−8.5 (−15.4 to −1.6; *p* = 0.015)	0.94 (0.87 to 0.97)	13.6	36.3
Narrowest part of the waist	−6.7 (−15.1 to 1.7; *p* = 0.118)	0.90 (0.79 to 0.95)	16.6	33.3
Widest part of the hips	−14.7(−23.1 to −6.3; *p* = 0.001)	0.89 (0.70 to 0.96)	16.6	97.8

## Appendix B

Table A2

**Table A2.** Picture mapping task: number of participants, percentage of participants, and 95% confidence intervals, for image width selected.

	Underestimated: percentage image shrunken (%)				Accurate	Overestimated: percentage image enlarged (%)			
Image width	84%	88%	92%	96%	100%	104%	108%	112%	116%
Number of participants (%)	0/65 (0.0)	3/65 (4.6)	7/65 (10.8)	13/65 (20.0)	17/65 (26.2)	8/65 (12.3)	9/65 (13.8)	7/65 (10.8)	1/65 (1.5)
95% confidence intervals	-	1.0 to 12.9	4.4 to 21.0	11.1 to 31.8	16.0 to 38.5	5.5 to 22.8	6.5 to 24.7	4.4 to 20.9	0.0 to 8.3
